# Motivating Factors and Psychosocial Barriers to Condom Use among out-of-School Youths in Dar es Salaam, Tanzania: A Cross Sectional Survey Using the Health Belief Model

**DOI:** 10.5402/2012/170739

**Published:** 2012-09-27

**Authors:** E. Katikiro, B. Njau

**Affiliations:** ^1^National AIDS Control Programme Division, Ministry of Health and Social Welfare, P.O. Box 11857, Dar es Salaam, Tanzania; ^2^Community Health Department, Kilimanjaro Christian Medical University College, P.O. Box 2240, Moshi, Tanzania

## Abstract

Condoms remain a cost-effective and relatively simple intervention to prevent HIV infection. However, condom use is still very low, particularly among youths aged 15 to 24. 348 individuals (186 males and 162 females) completed a pre-tested questionnaire. Logistic regression analysis was used to identify factors associated with condom use. Out of 348 respondents, 296 (85.0%) were sexually experienced, and 260 (87.8%) reported noncondom use in the past 3 months prior to the study. Among men, noncondom use was independently associated with feeling shy to buy condoms (AOR = 1.16; 95% CI 1.12–1.34), condoms reducing sexual pleasure (AOR = 8.19; 95% CI 3.98–17.01), and HIV is a serious and deadly disease (AOR = 0.36; 95% CI 0.28–0.46). Among women, experiencing forced sex (AOR = 1.16; 95% CI 1.10–2.78), condoms reduce sexual pleasure (AOR = 8.29; 95% CI 3.36–20.73), and inability to convince a partner to use condoms (AOR = 1.14; 95% CI 1.04–1.28) were predictors of noncondom use. In conclusion, sexually active youths in this population practice risky sexual behaviours, with low condom use practices. Strategies to improve condom use should address these psychosocial barriers associated with noncondom use.

## 1. Background

One of the current challenges on the prevention and control of HIV/AIDS faced worldwide is among youths aged 15–24 years. In fact, the global population of adolescents has reached over one billion, the largest in human history [1]. In Tanzania, almost two-thirds (65%) of the population is under the age of 24 and almost 20% are aged 15 to 24 [2, 3]. In fact, these young women and men are the future of Tanzania, and thus, an important age group for the growth and prosperity of the country.

Globally, it is estimated that 5.4 million young people aged 15 to 24 are living with HIV, 3.2 million of whom live in Sub-Saharan Africa [4]. In sub-Saharan Africa (SSA), youth lack access to HIV prevention education programes. For example, only 8 percent of out-of-school youth, have access to prevention education programes [5]. Overall HIV prevalence of 7.9% among youth aged 15 to 24 in Tanzania indicates that the HIV infection is two to three times higher among females than males (7% versus 3%), particularly in urban populations [2, 3]. Indicators of sexual activity among young people in Tanzania have revealed that a substantial proportion (46%) of unmarried youths aged 15–24 years is sexually experienced [2, 3]. A host of environmental, economic, and legal factors, together with social norms, are likely to influence early sexual debut and tendency for adolescents to be engaged in high-risk sexual behavior. In Tanzania, HIV prevention programes have been developed to target school youths and exclude out-of-school youths, increasing the risk of HIV infection among this risk age group [2, 6].

Several studies have documented the cost-effectiveness of condoms as a relatively simple intervention to prevent HIV infection [5]. However, studies on adolescent sexuality have shown a significant increase in sexual experiences at a younger age associated with inconsistent condom use [5, 7–9]. Several psychosocial and cultural barriers have been associated with noncondom use among youths aged 15 to 24 [6, 10, 11]. Moreover, more than 200 myths, misconceptions, and fears that may hinder access to or use of condoms have been noted [5]. A major challenge facing HIV prevention efforts among out-of-school youths aged 15 to 24 in Tanzania is inadequate research on factors that influence youth's intention to use condom. Studies conducted on factors associated with condom use among youths and adolescents in Tanzania were mainly on knowledge, attitudes, and practices (KAP), which did not use behavioral change models such as the Health Belief Model (HBM). This study therefore aims to identify factors that motivate or hinder condom use among out-of-school youths in an urban setting. Considering that the HBM is a behavioral change framework, which addresses several constructs influencing health behaviors (e.g., condom use), it is expected that the findings from this analysis will provide a basis for designing an effective HIV prevention programme for out-of-school youths aged 15–24 years in urban communities of Tanzania. 

## 2. Conceptual Framework 

**Figure 1:**
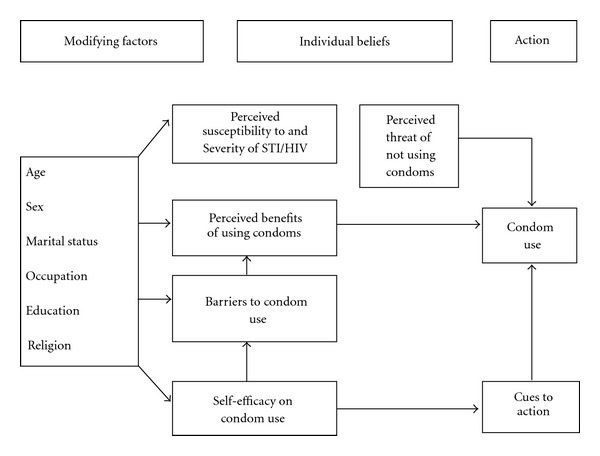
Operational description of the Health Belief Model.

This study, which was conducted in Kinondoni Municipality, Dar es Salaam, Tanzania used the Health Belief Model (HBM) as a conceptual framework to address the study objectives. The Health Belief Model postulates that an individual's actions are based on beliefs. According to the HBM, several factors, such as perceived susceptibility or vulnerability, perceived severity of an outcome or condition, perceived efficacy or benefits of a preventive measure, and the perceived barriers are important factors in decision making [12, 13] (see Figure 1). 

## 3. Methods

### 3.1. Study Setting

The study was conducted in Dar es Salaam, one of the 30 regions of Tanzania mainland, with a population of more than three million. Kinondoni Municipality is one of three municipal councils with a population of 1.4 million and youths aged 15 to 24 are estimated to be 234,003 (17.2%) [14, 15]. The HIV prevalence in Dar es Salaam in 2009 was estimated to be 9.3%, which is comparatively higher than the national prevalence of 5.9% [2, 3]. This study was done in Kinondoni municipality of Dar es Salaam's three municipal councils. Data was collected in 12 randomly selected wards (administrative areas) out of 27 wards. This study was conducted from April to May, 2010.

### 3.2. Sample and Sample Size Determination

The sample size was calculated using Epi Info version 6.0 statistical software. The result of a previous study conducted in Nigeria, which showed condom use among out-of-school youths to be 29% was used to calculate the sample size for the present study [16]. To detect a 10% difference in the rate of condom use with 95% confidence interval (CI) and 80% power, a sample of 316 was needed. With the addition of a 10% nonresponse rate, the final sample size became 348.

A multistage sampling method was applied in this study. A list of all wards in all four divisions within Kinondoni Municipality was used to randomly select 12 wards out of a total of 27 wards. Out of the 12 wards, an average of 29 households per ward was randomly selected to participate into the study. Using the local government register a list of all out-of-school 15–24-year-olds was made and stratified by age groups and gender. Proportional sampling was used to select equal numbers of eligible males and females for participation in the study. Consent was obtained after the potential participants and their parents (for those below 18 years) were informed of the study's objectives.

### 3.3. Ethical Clearance

Approval for this study was obtained from the Kilimanjaro Christian Medical University College Research Ethics Committee. Permission to conduct the study was sought from the Kinondoni Municipal authority.

### 3.4. Measurements

The survey questionnaire was designed as an adaptation from the instrument used in Benin for assessing key constructs of Health Belief Model related to condom use among youths aged 15–24 years [17]. Social demographic characteristics, proportions of condom use, and individual risk factors were also noted. A pretest of the tool was carried out on a convenience sample of 30 out-of-school youths of both genders for clarity and to ascertain internal consistency. Respondents were given a self-administered questionnaire in Kiswahili, a language well known to the participants. Confidentiality was assured by providing a private room or other place. Four trained research assistants (2 males; 2 females) who were about the same age (range 15–24 years old) and the same gender assisted those unable to read or write. The completed questionnaires were checked by the research assistants for errors and missing data before participants were allowed to go. 

The dependent variable for this study was measured by asking respondents if they have had sex without condom in the past 3 months (1 = yes, 2 = no). A response of “yes” indicated sexual risk behavior. 

Social-demographic characteristics included: age categorized into two groups (15–19 and 20–24), sex, marital status divided into two categories (married and unmarried), ability to read divided into 2 categories (able to read or read with difficulties), occupation was categorized into two groups (employed or unemployed), and religion. Behavioral risk factors included number of lifetime sexual partners, use of condoms in the first sexual contact, whether a partner has demanded unprotected sex, whether respondent had experienced forced sex, whether the respondent had had sex under the influence of alcohol, and whether the respondent had ever had sex under the influence of substances. Each of the questions required a response of 1 = yes, 2 = no.

Psychosocial barriers to condom use were assessed with the following questions: My religion prohibits condom use, I do not like condoms, condoms reduce sexual pleasure, condoms offer no protection, and I feel shy to buy condoms. Each of the questions required a response of 1 = yes, 2 = no. 

Motivational factors to condom use were assessed by the following questions: whether respondents perceived HIV as a severe and deadly disease, perceived benefits of condom to prevent STIs/HIV, whether respondent feels confident to discuss condom use prior to sexual intercourse, and whether respondent is able or not to convince a partner to use condoms. Each of the questions required a response of 1 = yes, 2 = no. 

### 3.5. Statistical Analysis

Data were edited, cleaned, coded, entered, and analysed using Statistical Package for Social Sciences version-12.0.1 (SPSS for Windows; SPSS, Chicago, IL, USA). The assessment included descriptive and multivariate analyses. Probability values (*P* values) were calculated at the 0.05 level of significance, odds ratios (OR) and 95% confidence intervals (CI) were provided in bivariate analysis. Two-sided chi-square tests for association were computed to detect differences in categorical variables and logistic regressions were calculated when testing the relationships between categorical variables. To identify independently associated factors, two logistic regression models (women and men) were performed, with noncondom use in the past 3 months as the outcome variable. All explanatory variables that were associated with the outcome variable in bivariate analysis, variables with a *P*-value of ≤0.05 were included in the logistic models. 

## 4. Results

### 4.1. Characteristics of Respondents

The study was based on a sample of 348 randomly selected respondents. A total of 186 men and 162 women respondents were interviewed, yielding a response rate of 100%. The mean age for men was 20.37 ± 2.58 (mean ± SD) years while it was 19.56 ± 2.60 (mean ± SD) for women. The sociodemographic characteristics are summarized in Table 1.

### 4.2. Behavioral Risk Factors

Two hundred and ninety six participants reported had had sex in the past 3 months prior to the study. Out of 296 sexually active participants, 260 (87.8%), reported noncondom use in the past 3 months prior to the study. Males were marginally more likely to use a condom compared to their female counterparts (41.3% versus 36.7%). In addition, females were more likely to have unprotected sexual intercourse due to a demand from a sexual partner compared to males (53.7% versus 38.7%; *P* < 0.01). Furthermore, the study revealed that more females than males experienced forced sex (44.9% versus 18.7%; *P* < 0.001). There were no significant differences between males and females in the other behavioral risk factors (data not shown).

### 4.3. Psychosocial Barriers to Condom Use

As depicted in Table 2, there was significant difference in perceived psychosocial barriers to condom use among condom users (*n* = 36) and noncondom users (*n* = 260) in the past 3 months prior to the study. Participants who agreed that their religion prohibits condom use were more likely to report having sex without a condom (odds ratio (OR) = 5.08; 95% CI 2.22–11.86; *P* < 0.001). In addition, sex without a condom was associated with a belief that condoms reduced sexual pleasure (OR) = 15.00; 95% CI 5.58–41.87; *P* < 0.001), condoms offer no protection (OR) = 7.83; 95% CI 3.32–18.95; *P* < 0.001), and feeling shy to buy a condom (OR) = 25.78; 95% CI 13.27–36.94; *P* < 0.001).

### 4.4. Determinants of Condom Use

Table 3 presents a summary of selected determinants of behavioral change associated with noncondom use among male and female participants in the past 3 months prior to the study. In the bivariate analyses for men, five variables (experiencing forced sex, demanded unprotected sex by a partner, feeling shy to buy condoms, condoms reducing sexual pleasure, and perceiving HIV as a serious and deadly disease) were found to be associated with noncondom use. Males who had experienced forced sex were more likely to report sex without a condom compared to those who had never experienced forced sex (odds ratio (OR) = 1.66; 95% CI 1.12–2.35; *P* = 0.02). Additionally, males who were asked not to use condoms by a partner were more likely to report sex without a condom (OR = 1.58; 95% CI 1.10–2.27; *P* = 0.01). Males who agreed that they felt shy to buy condoms were more likely to report sex without a condom compared to those who did not feel shy (OR = 1.12; 95% CI 1.10–1.24; *P* = 0.001). Furthermore, males who perceived that condoms reduce sexual pleasure were more likely to report sex without a condom compared to those who did not perceive so (OR = 1.20; 95% CI 1.13–1.27; *P* = 0.001). On the other hand, males who perceived HIV as a serious and deadly disease were less likely to report sex without a condom compared to those who did not perceive so (OR = 0.40; 95% CI 0.18–0.63; *P* < 0.001).

For females, seven variables (experiencing forced sex, feeling shy to buy condoms, condoms reducing sexual pleasure, cannot convince partner to use a condom, prior discussion about condom use, perceiving HIV as a serious and deadly disease and having sex while drunk,) were found to be significantly associated with condom use. Females who had experienced forced sex were more likely to report sex without a condom compared to those who had not (OR = 1.43; 95% CI 1.14–2.77; *P* = 0.02). In addition, females who agreed that they felt shy to buy condoms were more likely to report sex without a condom compared to those who did not feel shy (OR = 1.30; 95% CI 1.05–1.17; *P* = 0.001). Furthermore, females who perceived that condoms reduce sexual pleasure (OR = 1.27; 95% CI 1.05–1.54; *P* = 0.001), or that they cannot convince a partner to use condoms (OR = 1.10; 95% CI 1.02–1.18; *P* = 0.02) were more likely to report sex without a condom. Females who discussed condom use prior to having sex were less likely to report sex without a condom compared to those who never had prior discussion (OR = 0.57; 95% CI 0.49–0.67; *P* < 0.001). Lastly, perceiving that HIV is a serious and deadly disease (OR = 0.51; 95% CI 0.33–0.78; *P* < 0.001) and having sex while drunk (OR = 0.39; 95% CI 0.20–0.75; *P* = 0.001) were negatively associated with condom use among females participants. 

In the multivariate logistic regression analyses for men, three variables (feeling shy to buy condoms, condoms reducing sexual pleasure, and perceiving HIV as a serious and deadly disease), were found to be associated with noncondom use (Table 3). Males who were shy to buy condoms had an increased odd to report sex without a condom, in comparison to those who were not shy (adjusted odds ratio (AOR) = 1.16; 95% CI 1.12–1.34). Among male respondents, the strongest predictor of having sex without a condom was the perception that condoms reduce sexual pleasure (AOR = 8.19; 95% CI 3.98–17.01). On the other hand, males' respondents who perceived that HIV is a serious and deadly disease were less likely to report having sex without a condom compared to those who did not perceive so (AOR = 0.36; 95% CI 0.28–0.46).

For females, six variables (having sex while drunk, experiencing forced sex, condoms reduce sexual pleasure, cannot convince partner to use a condom, prior discussion about condom use and perceiving HIV as a serious and deadly disease) were found to be significantly associated with having sex without a condom. As depicted in Table 3, females who reported not having sex while drunk were less likely to report having sex without a condom compared to those who had sex while drunk (AOR = 0.16; 95% CI 0.13–0.21). Females who had experienced forced sex were more likely to report having sex without a condom (AOR = 1.16; 95% CI 1.10–2.78). Among female respondents, the strongest predictor of having sex without a condom was the perception that condoms reduce sexual pleasure (AOR = 8.29; 95% CI 3.39–20.73). Females who agreed that they cannot convince a partner to use condoms were more likely to report having sex without a condom than those who did not (AOR = 1.14; 95% CI 1.04–1.28).

Females who did not report prior discussion about condoms with their partners were 59% less likely to use a condom, in comparison with those who reported having prior discussion (AOR = 0.41; 95% CI 0.18–0.63). Lastly, females who perceived that HIV is a serious and deadly disease were less likely to report having sex without a condom than those who did not perceive HIV as a serious and deadly disease (AOR = 0.39; 95% CI 0.21–0.66).

## 5. Discussion

The aim of the study was to determine factors that motivate or hinder condom use among out-of-school youths aged 15 to 24 in an urban setting in Tanzania. The majority (85%) of out-of-school youths in this study reported engaging in premarital sex. In addition, 87.8% of the sexually experienced respondents reported having unprotected intercourse in the past 3 months, with male respondents marginally more likely to report condom use than their female counterparts. This finding substantiates national data gathered by the 2006-2007 Demographic Health Survey (DHS) for Tanzania, whereby 24% of unmarried youths aged 15–24 years reported unprotected intercourse [2] and may reflect a more fundamental problem that condom use decreases among adolescents as sexual experiences increases, despite of the high risk of HIV infection [2, 10]. This observation supports the need for an innovative community outreach HIV prevention services for out-of-school youths in Tanzania [5, 6]. This study used the Health Belief Model (HBM) to assess factors associated with condom use among the sample population. According to the HBM framework, perceived barriers to prevention are regarded as the single most powerful predictor for decision making [12, 13, 17]. Consistent with studies conducted elsewhere, the study findings confirmed that the psychosocial barriers are associated with noncondom use among sexually experienced respondents [5, 17, 18]. The psychosocial factors associated with noncondom use in this study were as follows: my religion prohibits condoms, condoms reduce sexual pleasure, condoms offer no protection, and feeling shy to buy a condom. The strongest psychosocial barrier to condom use, for both men and women was their concern regarding condoms' reduction of sexual pleasure. Respondents who perceived that a condom reduces sexual pleasure were more likely to report having sex without a condom when compared to those who did not perceive that a condom reduces sexual pleasure. Generally, adolescents tend to believe that a condom reduces sexual pleasure and this belief discourages condom use. According to existing literature on sexuality, there are four basic reasons why an individual may want to have sex. The first is sex for pleasure; the second is sex for procreation; the third is sex for money/or gifts; and the fourth is sex for cultural rituals [5]. The finding suggests that the interventions designed to increase condom use, should address the belief that condoms reduce pleasure among out-of-school youths. An intervention designed to increase motivation for condom use while addressing this salient barrier may be an effective approach [5, 11, 19–21]. 

Using the HBM to assess motivating factors associated with condom use among out-of-school youths, we found that perceiving HIV as a serious and deadly disease was strongly associated with condom use in both male and female models. Respondents who perceived HIV as a serious and deadly disease were less likely to report having sex without a condom when compared to those who did not perceive HIV as a serious and deadly disease. This finding supports one of the important constructs of the HBM, which postulates that perceived susceptibility and severity of a condition (e.g., HIV infection) or disease is an important component, which may induce action or inaction and emphasize that behavioral change will be successful, if people feel threatened by their current behavioral patterns [13]. There is thus a need to strengthen current strategies based on increasing perceived severity of HIV as a deadly disease to encourage condom use among out-of-school youths in this population.

Champion and Skinner observed that self-efficacy and cues for action are the key components of HBM framework and are important determinants for an individual in taking an action and the degree of persistence they would demonstrate in the face of failure and/or adversity [13, 22]. However, in this study self-efficacy was not predictive of condom use and thus was eliminated from the analysis. This observation is contrary to other studies in other settings, which observed an association between high self-efficacy and condom use. Self-efficacy, which is defined as a confidence in individual's capacity to perform a desired behavior was observed to be a driving force in consistent condom use [6, 17, 21]. Thus further research, perhaps with combined theoretical frameworks, such as social learning theories [23], may help to assess the association between self-efficacy and condom use among this population [19, 20, 22, 24].

In this study, discussion on condom use prior to having sex was associated with condom use among female participants. Females who reported to discuss condom use prior to having sex were less likely to report having sex without a condom when compared to those who did not discuss condom use. These findings are consistent with the HBM, which postulates that discussion on health behavior (e.g., condom use) can act as a trigger for action. From the HBM perspectives, human behavior depends on cues that elicit certain response and that maintenance of the desired behavior, such as condom use may occur by automation after sufficient repetition [13]. The finding suggests that perhaps a goal-oriented method of communication designed to potentiate readiness to condom use may be an effective approach [19]. 

Experiencing forced sex was independently associated with noncondom use among female respondents. Females who had experienced forced sex were more likely to report having sex without a condom when compared to those who had not experienced forced sex. In addition, female respondents who had sex while drunk were more likely not to use condoms. As expected, females who agreed to have sex while drunk were more likely to report having sex without a condom when compared to those who did not had sex while drunk. Although this observation does not support any construct of the HBM, it underscores the role of risky behaviors in understanding the progression towards health behavior change [19, 21, 25] and underscores the importance of addressing the risk behavior of mixing unprotected sexual intercourse and alcohol and skill development for safer sex among out-of-school youths. 

This study has certain limitations. First, the study asked respondents to recall events that may have happened in the distant past. Recall bias is more likely among older youths who may be unable to remember the exact timing of their first sexual intercourse and condom use. Second, the study was limited in that it relied on self-report. It is known that the self-reported behavior is subject to reporting bias. Third, the study did not assess the grades that out-of-school participants had completed. Future research among out-of-school participants needs to include this variable in order to identify the influence of educational attainment on behavioral change in this population. Fourth, HIV knowledge was not assessed in this study, which may have affected the study findings particularly in a setting where many myths related to HIV do exist. Item level analyses were limited due to the low internal reliability of items. Future studies needs to assess important constructs related to condom use attitudes and intention. Lastly, cross-sectional studies are not adequate for measuring the directionality of associations found and therefore this cannot account for potential confounders. Nevertheless, the study findings provide very important insight into perspectives of out-of-school youths on condom use in Kinondoni Municipality in Dar es Salaam, Tanzania. The findings emphasize the extent to which noncondom use remains an important issue and the need to focus additional attention on this issue, particularly among out-of-school youths.

## 6. Conclusions

In this study the majority of out-of-school youths were sexually experienced, while condom use was low. Furthermore, most sexually experienced participants had unprotected intercourse in the past 3 months prior to the study. This observation may reflect a fundamental problem of noncondom use as sexual experiences increases, irrespective of perceived risk of HIV infection among out-of-school youths in Tanzania. Our finding supported some of the HBM constructs, such as perceived barriers, perceived severity of HIV infection, and cues for action, but also highlighted constructs of other theoretical frameworks. Our findings confirmed that psychosocial barriers are associated with noncondom use in both men and women. This finding pinpoints the importance of addressing psychosocial barriers associated with noncondom use and suggests strategies which will increase motivation for condom use in this population. Perceiving HIV as serious and deadly disease was strongly associated with condom use and suggests that the fear of a disease outcome may influence a successful behavioral change. In addition, discussion on condom use prior to having sex was associated with condom use, particularly among women. These findings emphasize the importance of cues for action as triggers to potentiate readiness to action, such as condom use. On the other hand, self-efficacy on intention to use a condom was not predictive of condom use in this sample. This suggests that further research is required to determine the association between self-efficacy on intention to use a condom and condom use among out-of-school youths. Additionally, having sex while drunk was associated with noncondom use, particularly among females respondents. This observation underscores the importance of addressing the risk behavior of mixing unprotected sexual intercourse and alcohol among this population. In conclusion, therefore, it is important that HIV programme planners and policy makers understands the importance of focusing on identified HBM constructs that have the greatest probability of influencing condom use by providing accurate information about benefits of condoms and address gender norms and skills to overcome HIV risk behaviors in efforts to dispel psychosocial barriers to condom use among out-of-school youths in Tanzania.

## Figures and Tables

**Table 1:** Sociodemographic characteristics of respondents (*n* = 348).

Characteristics of the respondents	Total	Males	Females
	*N* = 348 (%)	*n* = 186 (53.4)	*n* = 162 (46.6)
Age group (years)			
15–19	146 (42.0)	68 (36.6)	78 (48.1)
20–24	202 (58.0)	118 (63.4)	84 (51.9)
Marital status			
Unmarried	260 (74.7)	142 (76.3)	118 (72.8)
Married	88 (25.3)	44 (23.7)	44 (27.2)
Ability to read			
Able to read	291 (83.6)	156 (83.9)	135 (83.3)
Read with difficulty/not at all	57 (16.4)	30 (16.1)	27 (16.7)
Occupation			
Employed	84 (24.1)	48 (25.8)	36 (22.2)
Unemployed	264 (75.9)	138 (74.2)	126 (77.8)
Religion			
Christian	201 (57.8)	106 (57.0)	95 (58.6)
Muslim	128 (36.8)	69 (37.1)	59 (36.4)
Other	19 (5.4)	11 (5.9)	8 (5.0)

**Table 2:** Psychosocial barriers to condom use among condom users and noncondom users in the past 3 months (*n* = 296).

	Sex without condom past 3 months		OR [95% CI]; *P* value
	Yes (*n* = 260)	No (*n* = 36)
My religion prohibits condom use			
Yes	172 (66.2)	10 (27.8)	1
No	88 (33.8)	26 (72.2)	5.08 (2.22–11.86), *P* < 0.001
I do not like condoms			
Yes	164 (63.1)	28 (77.8)	1
No	96 (36.9)	8 (22.2)	0.49 (0.20–1.18), *P* = 0.08
Condoms reduce sexual pleasure			
Yes	231 (88.8)	6 (16.7)	1
No	21 (11.2)	30 (83.3)	15.00 (5.58–41.87), *P* < 0.001
Condoms offer no protection			
Yes	188 (73.8)	9 (25.0)	1
No	72 (26.2)	27 (75.0)	7.83 (3.32–18.95), *P* < 0.001
I feel shy to buy condoms			
Yes	229 (88.1)	6 (16.7)	1
No	31 (11.9)	30 (83.3)	25.78 (13.27–36.94), *P* < 0.001

**Table 3:** Unadjusted odds ratios (OR) and adjusted odds ratios (AOR) from logistic regression analyses that examined the association between selected determinants of behavioural change and noncondom use in past 3 months by gender (*n* = 296).

Variables	^ a^Males (*n* = 160)			^ b^Females (*n* = 136)		
Bivariate analysis	*P* value	OR	95% CI	*P*-value	OR	95% CI

Had sex while drunk	0.480	0.87	0.58–1.30	<0.001^†^	0.39	0.20–0.75
Experienced forced sex	<0.01*	1.66	1.12–2.35	<0.01*	1.43	1.14–2.77
Demanded unprotected sex from partner	<0.01*	1.58	1.10–2.27	0.765	0.94	0.60–1.45
I feel shy to buy condoms	<0.001^†^	1.12	1.10–1.24	<0.001^†^	1.30	1.05–1.17
Condoms reduce sexual pleasure	<0.001^†^	1.20	1.13–1.27	<0.001^†^	1.27	1.05–1.54
Cannot convince partner to use condoms	0.063	0.94	0.84–1.06	<0.01*	1.10	1.02–1.18
Discussed condom use prior to sex	0.521	0.91	0.82–1.95	<0.001^†^	0.57	0.49–0.67
HIV is a serious and deadly disease	<0.001^†^	0.40	0.18–0.63	<0.001^†^	0.51	0.33–0.78

Multivariate analysis^§^	*P*-value	AOR	95% CI	*P*-value	AOR	95% CI

Had sex while drunk	0.520	0.91	0.62–1.34	<0.001^†^	0.16	0.13–0.21
Experienced forced sex	0.574	1.14	0.73–1.76	<0.01*	1.16	1.10–2.78
I feel shy to buy condoms	<0.001^†^	1.16	1.12–1.34	0.58	0.78	0.29–2.10
Condoms reduce sexual pleasure	<0.001^†^	8.19	3.98–17.01	<0.001^†^	8.29	3.39–20.73
Cannot convince partner to use condoms	0.071	0.92	0.76–1.16	<0.01*	1.14	1.04–1.28
Discussed condoms use prior to sex	0.561	0.45	0.11–1.05	<0.001^†^	0.41	0.18–0.63
HIV is a serious and deadly disease	<0.001^†^	0.36	0.28–0.46	<0.001^†^	0.39	0.21–0.66

^†^ *P* < 0.001; *P < 0.01.

^a^2 Log likelihood test: 176.5; *R* ^2^ .224; ^b^2 Log likelihood test: 138.5; *R* ^2^ .257.

^§^Adjusted for sociodemographic, risky behaviours, psychosocial and motivating variables.
